# Supplementary material for: A machine-learned spin-lattice potential for dynamic simulations of defective magnetic iron
Source: Sci Rep. 2022 Dec 27;12:22451. doi: 10.1038/s41598-022-25682-5 (PMC9794737; doi:10.1038/s41598-022-25682-5)
Supplement: Supplementary file 1 — Supplementary Information. [file 41598_2022_25682_MOESM1_ESM.pdf]

A Machine-Learned Spin-Lattice Potential for Dynamic Simulations of Defective Magnetic Iron: Supplementary Materials

Jacob B. J. Chapman[1,*] **and Pui-Wai Ma**[1,+]

[1]United Kingdom Atomic Energy Authority, Culham Science Centre, Abingdon, Oxfordshire, OX14 3DB, United Kingdom
[*]Jacob.Chapman@ukaea.uk
[+]Leo.Ma@ukaea.uk

ABSTRACT

In this Supplementary Materials to the manuscript "A Machine-Learned Spin-Lattice Potential for Dynamic Simulations of Defective Magnetic Iron", we discuss the (1) fitting procedure of our machine-learned spin-lattice potential (MSLP) for iron, (2) details of the implementation of MSLP to the spin-lattice dynamics code SPILADY, and (3) addition numerical results.

1 Fitting Procedure

1.1 Data Selection

An artificial neural network (ANN) can be considered as an universal approximator if unbounded non-linear activation functions are used[1]. It is highly flexible and can be adopted to various type of problems. However, ANNs are poor at extrapolation. It can hardly produce sensible output if the input is very different from data in the training set. The inability to extrapolate is a fundamental issue of ANNs[2]. As such, the quality of an ANN depends upon the data to which it is trained. This presents two limitations[3]:

1. The accuracy of the model's prediction cannot exceed the accuracy of the training data.
2. The transferability of a model is bound by the phase space represented in the training data.

Any noisy, incorrect, and/or redundant training data would inevitably hinder the ability of a machine-learned model to accurately learn the underlying relationships between the effective coordinates and target values. Consequently, well selected data is essential to produce an accurate and transferable model[4,5].

A major difference of machine-learned interatomic potential for molecular dynamics and spin-lattice dynamics arises from the extra magnetic degrees of freedoms. To assist the potential to learn the magnetic and non-magnetic contribution in stages, we generated two sets of complementary data, where one is with non-magnetic and the other is with magnetic configurations. Non-magnetic configurations are produced using non-spin-polarised density functional theory (DFT). Magnetic configurations are produced mainly from spin-polarised DFT calculation, which only considers collinear magnetic moment. In addition, some samples in non-collinear magnetic configurations were generated using the constraint method[6].

Since our aim is to produce a machine-learned spin-lattice potential (MSLP) for defective magnetic iron, which is capable of simulating the dynamic evolution of defects induced by irradiation, it requires data covering both perfect and distorted structures, as well as many different magnetic configurations. We tried to generate a wide range of data that hopefully can cover a significant part of the phase space. The training data set now includes ideal crystal structures with varying magnetic states for BCC, FCC and HCP phases, deformed structures representing displacements of atoms from their ideal lattice sites, strained perfect and deformed structures with varying lattice parameters, simple defects including vacancies and self-interstitial atoms, as well as deformations of both the transverse and longitudinal components of the magnetic moments for various structural configurations.

All training data was calculated using DFT via the VASP package[7–10]. For bookkeeping and automation, we group configurations into distinct classes, subcategorized by their magnetic order. DFT simulation details and outputs were collected and stored in an SQLite database. The classes are:

- **Class 1**: Bulk and deformed primitive unit cells of BCC, FCC, HCP, C15 and A15 phases. Deformed cells are constructed by applying a deformation tensor **D** to introduce various strains to the perfect cells.

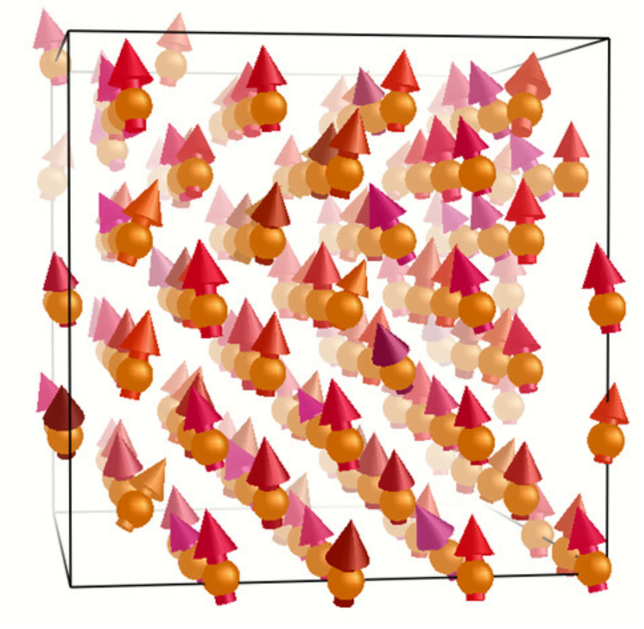

Figure 1. Snapshot of the spin dynamics simulation of ferromagnetic α-Fe at 100K. It demonstrates at finite temperatures magnetic excitations are realized as non-collinear orientations of magnetic moments. The system evolves according to Langevin spin dynamics using the Ma-Dudarev potential[14].

- **Class 2**: Epitaxial BCC-BCT-FCC Bain distortion[11] at constant volume.
- **Class 3**: Small random displacement of atoms from ideal lattice sites in BCC and FCC phases ($|\Delta \mathbf{r}_i| \leq 0.02$ Å).
- **Class 4**: Snapshots of atomic configurations of perfect crystal produced by MD simulations. MD simulations using Derlet-Dudarev potential[12] were performed in an NPT ensemble at 10K, 100K, 200K, 300K and 800K.
- **Class 5**: Vacancy clusters with number of vacancy $n = 1,2,3,4$ (V_{Fe}^n) in simulation boxes using ideal lattice constant.
- **Class 6**: Self-interstitial atom (SIA) configurations in the BCC phase using ideal lattice constants, including $\langle 110 \rangle$, tetrahedral, $\langle 111 \rangle$, octahdral and $\langle 100 \rangle$ SIA configurations.
- **Class 7**: Snapshots of atomic configurations of SIA and mono-vacancy configurations produced by MD simulations. MD simulations were performed at low temperature allowing exploration of the energy well.
- **Class 8**: Amorphous configurations. It is produced by quenching systems at liquid states through conjugate gradient method, where liquid states were produced by MD using Derlet-Dudarev potential.[12].
- **Class 9**: Primitive BCC and FCC unit cells with different values of magnetization. The magnetization of the simulation box is controlled by setting the difference between the number of up and down electrons, which is controlled by the keyword "NUPDOWN" in VASP.
- **Class 10**: Snapshots of non-collinear magnetic configurations on perfect BCC lattice produced by spin dynamics simulations. Spin dynamics simulations were performed at 100K, 200K, 600K and 800K using Langevin spin dynamics[13] and Ma-Dudarev potential[14]. An example of magnetic configuration is shown in Figure 1. Then, the non-collinear magnetic configurations were generated using constrained method for non-collinear DFT[6].
- **Class 11**: Small random non-collinear displacements of magnetic moments in BCC and FCC phases.

1.2 Density Functional Theory Calculations

All training data was generated using the VASP package[7–10]. For most calculations, we performed non-spin-polarized[15,16] or spin-polaried[17] DFT calculations. We used the projector augmented wave (PAW) pseudopotentials[18–20] which includes the

$3p$ states as valence states, with a total of 14 valence electrons. The plane wave cut off energy was set to 400 eV. Exchange-correlation energy was approximated using the GGA Perdew-Burke-Ernzerhof (PBE) functional[21,22]. Brillouin zones were sampled using the Monkhorst Pack scheme such that the smallest spacing between k-points is 0.01Å^{-1}. Convergence criteria of the self consistent field iteration was set to 10^{-8} eV. Forces were relaxed to a convergence of 10^{-4} eV/Å for bulk-like systems and 10^{-3}eV/Å for simulation cells containing defects.

In order to sample magnetic configurations in excited states, we performed non-collinear magnetic configurations using constrained method[6]. To constrain the orientations of magnetic moments to chosen directions $\{\mathbf{e}_I\}$, one can apply a energy penalty term E_p to the total energy functional:

$$\begin{aligned} E &= E_0 + E_p, && (1)\\ &= E_0 + \sum_I^N \lambda(|\mathbf{M}_I^F| - \mathbf{e}_I \cdot \mathbf{M}_I^F), && (2) \end{aligned}$$

where λ is a Lagrange multiplier which acts to change the degree of enforcement of the constraint on site I, and E_0 is the DFT energy. The E_p acts to introduce an effective magnetic field to each atom. The E_p is analytically proven to be inversely proportional to λ[6]. A large enough λ is chosen such that the change of E_p is smaller than 1meV.

Due to the use of PAW method, there is ambiguity on the definition of atomic magnetic moment. One may define the magnetic moment of atom I through the spatially varying magnetic density $\mathbf{m}(\mathbf{r})$:

$$\mathbf{M}_I^F = \int_{\Omega_I} \mathbf{m}(\mathbf{r}) F_I(|\mathbf{r} - \mathbf{r}_I|) d\mathbf{r}, \tag{3}$$

where the radius of the sphere Ω_I has been chosen to match the VASP parameter RWIGS of the PAW psuedopotentials which for our calculations is 1.131a$_0$. This satisfies that spheres are large enough but not overlapping. To prevent a discontinuity at the boundary of the constraint field, a smoothing function $F_I(\mathbf{r} - \mathbf{r}_I) = \sin x/x$ and $x = \pi|\mathbf{r} - \mathbf{r}_I|/R_I$ is introduced[6].

In our MSLP, the magnitude of magnetic moment can change representing the itinerant nature of electrons. However, in the constrained non-collinear DFT calculations, the magnitude of magnetic moment is allowed to relax to achieve lowest energy subjecting to constrains. The information about the energy landscape corresponding to the change of magnitude is not captured here, but through other part of the training data.

1.3 The Loss Function

A single atomic configuration can provide the information of total energy, atomic forces, box stresses and atomic magnetic effective fields. We can design a Loss function that can capture all these information. The aim is to develop a model that can reproduce target data with sufficient accuracy whilst maintaining generality. The act of training is essentially to minimise the Loss function:

$$\mathscr{L} = \mathscr{L}_E + \mathscr{L}_F + \mathscr{L}_\sigma + \mathscr{L}_H + \mathscr{L}_{\text{reg}} \tag{4}$$

with respect to a parameter vector:

$$\mathbf{p} = (\{V^n\}, \{r_n^V\}, \phi, \{t_n\}, \{r_n^t\}, J_0, \{a_n\}, \{b_n\}, V_0, \{\mathbf{W}\}, \{\mathbf{b}\}). \tag{5}$$

The Loss function for energy:

$$\mathscr{L}_E = \frac{1}{2N_{\text{config}}} \sum_c^{N_{\text{config}}} w_c^E \left(\Delta E_c^2 + \Delta E_{f,c}^2\right), \tag{6}$$

where N_{config} is the number of configuration, w_c^E is weight for configuration c, ΔE_c and $\Delta E_{f,c}$ are the differences in energy and formation energy between the model and target data, respectively.

The implementation of DFT in VASP can only calculate the total energy of a system. Therefore, we can only compare the energy difference of a system. All DFT energies were adjusted by subtracting the energy of an isolated atom multiplying the number of atoms in particular system. Then, one can calculate the energy difference:

$$\Delta E_c = E_c^{\text{MSLP}} - E_c^{\text{DFT}}. \tag{7}$$

The E_c^{MSLP} is the sum of all the atomic energy of configuration c calculated by MSLP.

The formation energy of a configuration c is defined as:

$$E_{f,c}^\alpha = E_c^\alpha - \left(\frac{N_c}{N_{GS}}\right) E_{GS}^\alpha \tag{8}$$

where N_c and N_{GS} are the number of atoms in configuration c and the corresponding ground state configuration, E^{α}_{GS} is the total energy of the ground state configuration, superscript α represents MSLP or DFT. Then, the difference of the formation energy:

$$\Delta E_{f,c} = E^{\mathrm{MSLP}}_{f,c} - E^{\mathrm{DFT}}_{f,c}. \tag{9}$$

The weight of configuration c is a function of ΔE_c, such that:

$$w^E_c(\Delta E_c) = w^E_{c0}\frac{1}{\log(0.01\Delta E_c^2 + 1.01)}, \tag{10}$$

where w^E_{c0} is a constant that assigned to a class or subclass of configurations. The design of this weight function is to automate the reduction in relative importance for configurations in the class whose energies are higher than the smallest energy in the set. This biases the loss function to find solutions which fit configurations near the minima against those in phase space which are unfavorable or unlikely.

The Loss function for atomic forces:

$$\mathscr{L}_F = \frac{1}{3N_{\mathrm{config}}}\sum_c^{N_{\mathrm{config}}}\frac{w^F_c}{N_c}\sum_i^{N_c}\left|\mathbf{F}^{\mathrm{MSLP}}_i - \mathbf{F}^{\mathrm{DFT}}_i\right|^2, \tag{11}$$

where w^F_c is the weight, $\mathbf{F}^{\mathrm{MSLP}}_i$ and $\mathbf{F}^{\mathrm{DFT}}_i$ are atomic forces calculated by MSLP and DFT, respectively.

The Loss function for the stress of simulation cell:

$$\mathscr{L}_\sigma = \frac{1}{9N_{\mathrm{config}}}\sum_c^{N_{\mathrm{config}}} w^\sigma_c \sum_{i,j}\left|\sigma^{\mathrm{MSLP}}_{ij} - \sigma^{\mathrm{DFT}}_{ij}\right|^2, \tag{12}$$

where w^σ_c is the weight, $\sigma^{\mathrm{MSLP}}_{ij}$ and $\sigma^{\mathrm{DFT}}_{ij}$ are the stresses that a simulation box experiences. The calculation of $\sigma^{\mathrm{MSLP}}_{ij}$ is according to the Virial theorem.

The Loss function for magnetic effective field:

$$\mathscr{L}_H = \frac{1}{N_{\mathrm{config}}}\sum_c^{N_{config}}\frac{w^H_c}{N_c}\sum_i^{N_c}\left|\mathbf{H}^{\mathrm{MSLP}}_i - \mathbf{0}\right|^2, \tag{13}$$

where w^H_c is the weight, $\mathbf{H}^{\mathrm{MSLP}}_i$ is the magnetic effective field calculated by MSLP. In adiabatic self-consistent field DFT calculations, the effective field for each atom must be zero, if no external field is applied. For those training data generated using constrained method, we set the weight w^H_c to zero. It is because we cannot obtain the effective fields from DFT without ambiguity.

In the Loss function, we introduced a Ridge (or Tikohnov) regularisation term:

$$\mathscr{L}_{\mathrm{reg}} = \lambda\sum_{k\in\mathbf{p}} k^2. \tag{14}$$

It is to prevent overfitting, loss of generalisation or the model becoming too dependent on any particular free parameter. A fixed regularisation value of $\lambda = 10^{-6}$ is used.

1.4 Validation Procedure and Limitations

Once the conventional spin-lattice potential had been trained (steps 1 and 2 in the training workflow), we began training neural networks of different sizes. One of the most computationally expensive steps is the calculation of the descriptors so we opted to minimise the input layer of the networks. To reduce cost, we reuse the G2 descriptors when building the magnetic descriptors (see Methods in article). As such, for all parameterisation tests using n G2 descriptors, our input layer would consist of $4n$ nodes. We found $n > 5$ with equidistantly spaced Gaussians in the range $[2, R_{cut}]$ could train reasonable networks when used with one or more hidden layers wider than 5 nodes. An input layer of $n = 9$ was determined to be large enough to build a sufficient representation of the configurational and magnetic environments, whilst small enough to reduce excessive computational cost.

In general, deeper neural networks were found to be slower to train whilst also overfitting the training data. This was compounded by the IPFIT codes inability to perform early stopping by simultaneously analysing a test set. As such, we would generally rely on the dynamic simulations used to calculate the Curie temperature to test for over fitting. An over fitted potential would have large variations in observable properties during a Langevin dynamics run at constant temperature and pressure (NPT ensemble). A small test set of approximately 400 unseen non-equilibrium configurations generated through *ab initio* molecular dynamics was also used to qualitatively assess the quality of a fit once converged (see Figure 2(ii)). The presented parameterisation relied only on a single hidden layer (36 x 9 x 1) to produce accurate results whilst remaining scalable to over 1 million atoms. Future updates of IPFIT will introduce improved testing and validation techniques such that deeper networks may be trained more reliably.

2 Atomic Force and Magnetic Effective Field

Details of spin-lattice dynamics that allows the change of magnitudes of magnetic moments[23] can be found in a review article[24] and related works[25,26]. An important part in the integration of equation of motion is the calculation of atomic force and magnetic effect field. They are derivatives of the Hamiltonian $\mathscr{H}$. The Hamiltonian of our MSLP is written as:

$$\begin{aligned}\mathscr{H} &= V^{\rm NM}(\mathscr{R}) + V^{\rm HL}(\mathscr{R},\mathscr{M}) + V^{\rm NN}(\mathscr{R},\mathscr{M}) \quad (15)\\ &= \sum_i F(\rho_i) + \frac{1}{2}\sum_i\sum_{j,j\neq i} V_{ij}(r_{ij}) - \frac{1}{2}\sum_i\sum_{j,j\neq i} J_{ij}(r_{ij})\mathbf{M}_i\cdot\mathbf{M}_j + \sum_i\left(A(\rho_i)\mathbf{M}_i^2 + B(\rho_i)\mathbf{M}_i^4 + C\mathbf{M}_i^6\right) + V_0^{\rm NN}\sum_i \mathscr{N}_i \quad (16)\end{aligned}$$

where $\mathscr{N}_i = \mathscr{N}(\mathbf{x}_i^0;\{\mathbf{W},\mathbf{b}\})$ is a trained neural network, $\mathbf{x}_i^0 = \{\{G_{i,h}\},\{G^H_{i,h}\},\{G^A_{i,h}\},\{G^B_{i,h}\}\}$ is a vector of descriptors for atom i. The functional form of the descriptors are discussed in the section of Method in the main text. For simplicity, we write $\mathbf{x}_i^0 = \mathbf{G}_i(\mathscr{R})$. Since nine G2 descriptors were used, there are totally $4\times 9 = 36$ elements in $\mathbf{G}_i$.

The atomic force on atom k is defined as:

$$\mathbf{F}_k = -\frac{\partial\mathscr{H}}{\partial\mathbf{r}_k} = -\sum_j \frac{\partial\mathscr{H}}{\partial r_{jk}}\hat{\mathbf{r}}_{jk}, \quad (17)$$

where

$$\begin{aligned}\frac{\partial\mathscr{H}}{\partial r_{jk}} &= \frac{\partial F(\rho_j)}{\partial\rho_j}\frac{\partial\rho_j}{\partial r_{jk}} + \frac{\partial F(\rho_k)}{\partial\rho_k}\frac{\partial\rho_k}{\partial r_{jk}} + \frac{\partial V_{jk}}{\partial r_{jk}}\\ &- \frac{\partial J_{jk}}{\partial r_{jk}}\mathbf{M}_j\cdot\mathbf{M}_k + \frac{\partial A(\rho_j)}{\partial\rho_j}\frac{\partial\rho_j}{\partial r_{jk}}\mathbf{M}_j^2 + \frac{\partial A(\rho_k)}{\partial\rho_k}\frac{\partial\rho_k}{\partial r_{jk}}\mathbf{M}_k^2 + \frac{\partial B(\rho_j)}{\partial\rho_j}\frac{\partial\rho_j}{\partial r_{jk}}\mathbf{M}_j^4 + \frac{\partial B(\rho_k)}{\partial\rho_k}\frac{\partial\rho_k}{\partial r_{jk}}\mathbf{M}_k^4\\ &+ V_0^{\rm NN}\left(\frac{\partial\mathscr{N}_j}{\partial\mathbf{G}_j}\frac{\partial\mathbf{G}_j}{\partial r_{jk}} + \frac{\partial\mathscr{N}_k}{\partial\mathbf{G}_k}\frac{\partial\mathbf{G}_k}{\partial r_{jk}}\right). \quad (18)\end{aligned}$$

The magnetic effective field is

$$\mathbf{H}_k = -\frac{\partial\mathscr{H}}{\partial\mathbf{M}_k}, \quad (19)$$

where

$$\begin{aligned}\frac{\partial\mathscr{H}}{\partial\mathbf{M}_k} &= -\sum_j J_{jk}\mathbf{M}_j + \left(2A(\rho_k) + 4B(\rho_k)M_k^2 + 6CM_k^4\right)\mathbf{M}_k\\ &+ V_0^{\rm NN}\sum_{j,j\neq k}\sum_h\left(\frac{\partial\mathscr{N}_j}{\partial G^H_{j,h}}G_{jk,h}\mathbf{M}_j + \frac{\partial\mathscr{N}_k}{\partial G^H_{k,h}}G_{kj,h}\mathbf{M}_j + 2\frac{\partial\mathscr{N}_j}{\partial G^A_{j,h}}G_{kj,h}\mathbf{M}_k + 4\frac{\partial\mathscr{N}_j}{\partial G^B_{j,h}}G_{kj,h}M_k^2\mathbf{M}_k\right) \quad (20)\end{aligned}$$

3 Additional Results

3.1 Quality of the MSLP

To evaluate the quality of our potential, we calculated the mean absolute error which quantifies the difference between the energy of the target DFT data and the energy predicted by the MSLP of the same configurations. Fig. 2a plotted the DFT energies against the predicted energies of all configurations used for training. The mean absolute error for all data is 46.3 meV/atom. We note this error is larger than most machine-learned potentials which are within 20meV/atom[27,28]. However, we should note the additional $3N$ degrees of freedoms due to magnetism in our model.

If we examine the mean absolute error according to classes in the training data, the mean absolute errors for classes 1 to 11 are 18.4, 49.3, 4.26, 2.29, 1.67, 0.70, 8.26, 31.6, 76.9, 22.15 and 8.46 meV/atom, respectively. For configurations near stable and metastable states, the mean absolute error is small and comparable to other machine-learned potentials for MD. The largest error comes from class 9, where energies were calculated subjecting to constraint controlling the value of magnetization. It is understandable that the mean absolute error is large in this data set, because most data contribute little to the energy loss function according to the functional form of w_c^E, i.e. weight is small when energy is high, where the data set has the largest energy range. The second largest error is from Class 2. We found the largest discrepancy comes from the antiferromagnetic BCC configurations. At full relaxed conditions, its DFT energy is 0.46 eV/atom higher than the ferromagnetic BCC configuration. If we consider the melting temperature of iron $T_{\rm melt} = 1811$K, we know the corresponding energy scale is about $k_B T_{\rm melt} = 0.16$eV. Such discrepancy should have little consequence in applications.

Table 1. Formation energies of self-interstitial atom and mono-vacancy configurations in FM BCC Fe calculated using DFT and MSLP. DFT data is labelled to distinguish different DFT paradigms. Current DFT calculations using VASP with semi-core PAW pseudopotential and planewave basis set. Chapman *et al.*[29] performed calculations using OpenMX with basis composed of a linear combination of psuedoatomic orbitals (PAOs). Olsson *et al.*[30] performed calculations using VASP with conventional PAW pseudopotentials and ultrasoft psudopotentials (USPP). Fu *et al.*[31] performed calculations using SIESTA with a basis set of PAOs. The formation energy of defects calculated using MSLP adopted the atomic positions and magnetic moments obtained from VASP-PAW-SC calculations. Results are in good agreement with the presented DFT calculations.

	Formation Energy (eV)					
Defect	MSLP	VASP-PAW-SC	OpenMX-PAO[29]	VASP-PAW[30]	VASP-USPP[30]	SIESTA-PAO[31]
⟨110⟩	4.35	4.29	4.49	4.02	3.94	3.64
Tetrahedral	4.89 (0.54)	4.78 (0.49)	4.98 (0.49)	4.44 (0.42)	4.46 (0.52)	4.26 (0.62)
⟨111⟩	5.02 (0.67)	5.09 (0.80)	5.26 (0.80)	4.72 (0.70)	4.66 (0.72)	4.34 (0.70)
⟨100⟩	5.65 (1.30)	5.53 (1.24)	5.58 (1.10)	5.13 (1.11)	5.04 (1.10)	4.64 (1.00)
Octahedral	5.80 (1.45)	5.67 (1.38)	5.74 (1.25)	5.29 (1.27)	5.25 (1.31)	4.94 (1.30)
Vacancy	2.09	2.17	2.18	2.15	2.02	2.07

A difficult point of producing a sensible potential is to guarantee the ground state ferromagnetic BCC configuration always attains the lowest energy. It is achieved through using the functional from of w_c^E, which amplified the importance of data for configurations with an energy close to the ground state. We can see in Fig. 2 that the lowest energy configuration in DFT data is the lowest energy configuration predicted by MSLP. We also plotted the forces and stresses calculated using DFT against MSLP in Fig. 2. They show good correlations. The mean absolute errors of force and stress are 0.1 eV/Å and 2.4 GPa, respectively.

In Fig. 3 the energies of BCC and FCC phases in non-magnetic state and the most stable magnetic states are shown as a function of lattice constant. DFT calculations show ferromagnetic and double layer antiferromagnetic state are most stable in BCC and FCC phases, respectively. Magnetism can lower the energy of both BCC and FCC phases. Our MSLP reproduced such phenomenon quantitatively well.

Fig. 4 shows the change of energy of ferromagnetic BCC iron as a function of the magnitude of magnetic moments calculated using VASP and MSLP. It shows an energy well resembling Landau functional form, where the minimum is about 0.4 eV/atom lower than non-magnetic case. It causes the formation of spontaneous magnetic moment M_s. The magnitude can also fluctuate due to thermal excitation. We should note in our MSLP, the Hamiltonian is rotational symmetry, so the energy is independent of the direction of magnetization in ferromagnetic state. The position of the minimum and the barrier height are well reproduced for FM BCC. However, the shape of the well warrants refinement in future parameterisations. The incorrect profile of the region between $M = 0$ and $M = M_s$ partially accounts for the high error for Class 2 configurations in the database.

3.2 Point defects

Magnetic interactions are responsible for the anomalous order of SIA stability for BCC iron which supports a ⟨110⟩ orientation opposed to ⟨111⟩ found in other BCC transition metals. Various DFT calculations as show in Table 1 are compatible with each other concerning the stability of SIA configurations, where the energy of ⟨110⟩ < tetrahedral < ⟨111⟩ < ⟨100⟩ < octhahedral.

We performed conjugate gradient method to relax various SIA configurations using our MSLP. However, except the ⟨110⟩ dumbbell, which is the most stable configuration, none of them can relax to decided configuration. It is understandable because they are unstable configurations. In DFT, most codes would determine and impose symmetry of a system, and so restricted the relaxation of atomic positions. However, a molecular statics calculation does not impose any symmetry when relaxing a system, so high energy configuration is difficult to hold. Therefore, we calculated the formation energy of defects using the atomic positions and magnetic moments of configurations obtained from present VASP calculations without further relaxation. The formation energy calculated using MSLP are well compatibility with our DFT calculations and other works.

We performed a nudged elastic band calculation between the ⟨110⟩ and ⟨111⟩ dumbbell self-interstitial atom configurations in FM BCC iron. It is to check if there is any energy barrier preventing the transition of a ⟨111⟩ dumbbell to the lower energy ⟨110⟩ configuration. Simulation cells contain 251 atoms. To reduce computational cost, we used standard PAW pseudopotentials instead. we generated 5 images along the transition pathway. The tolerance of SCF cycles is set to 10^{-7}eV/step and force is set 10meV/Å. No energy barrier was detected between the two configurations. It essentially means small perturbation, such as thermal excitation, can drive a ⟨111⟩ dumbbell to relax into a ⟨110⟩. dumbbell configuration.

3.3 Dislocation Loop

In addition to the SIA loop that we presented in the main text, we also constructed a shear loop. We constructed a loop in a simulation box containing 128,000 atoms via the ATOMSK code[32] with the Burgers vector $\mathbf{b} = a_0[100]$ and normal $\mathbf{n} = [001]$.

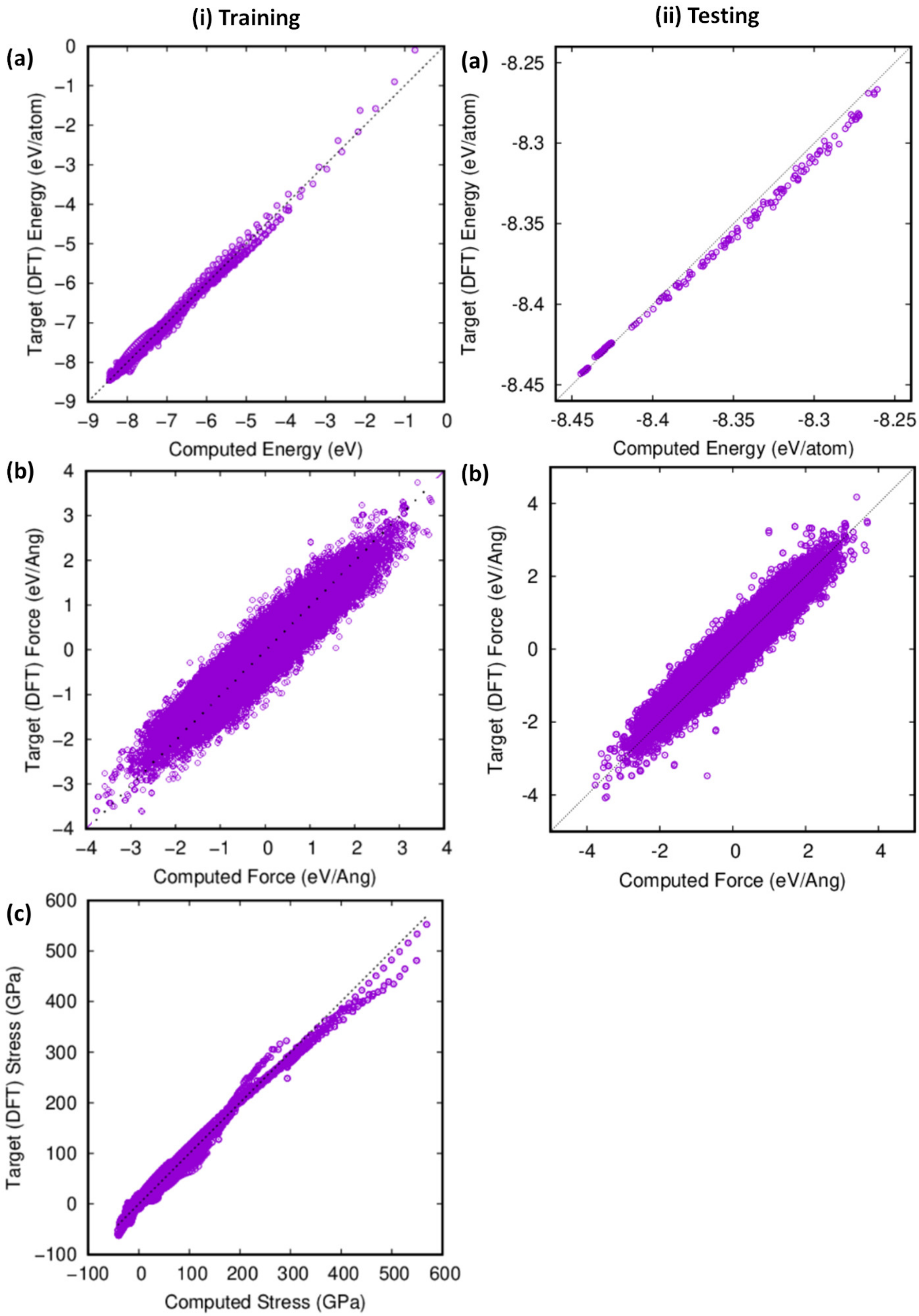

Figure 2. Comparison between DFT and MLSP model (a) energies (b) forces and (c) stresses (c) for all configurations supplied in the training (i) and test (ii) databases.

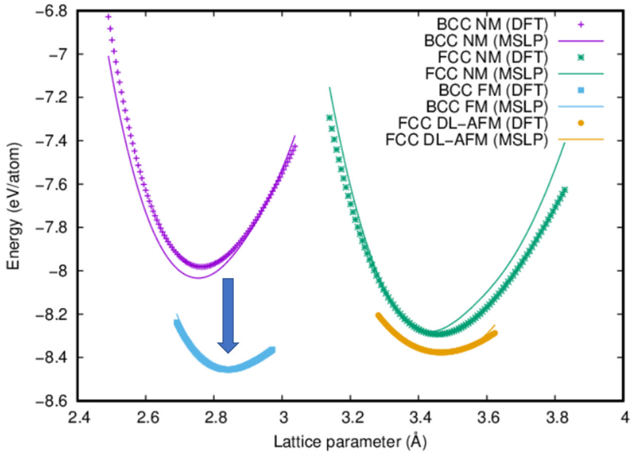

Figure 3. The energy of non-magnetic (NM) BCC, ferromagnetic (FM) BCC, non-magnetic (NM) FCC and double layer antiferromagnetic (DL-AFM) FCC iron as a function of lattice parameter calculated using VASP and MSLP. In non-magnetic case, FCC phase has lower energy than BCC phase. Magnetism lowers the energy of the BCC phase making it the most stable state.

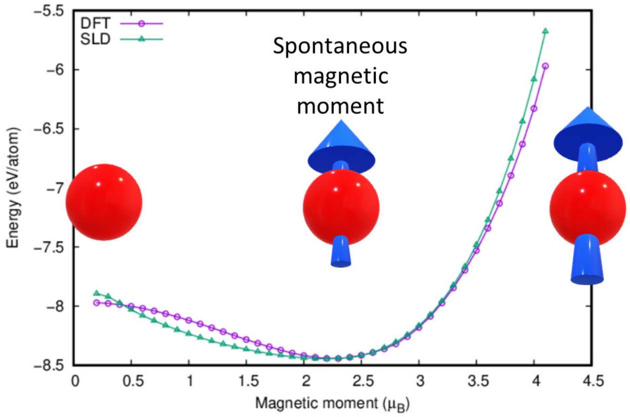

Figure 4. Change of energy of bulk BCC iron as a function of the magnitude of the magnetic moments calculated using VASP and MSLP. A fixed lattice parameter a =2.83Å is used.

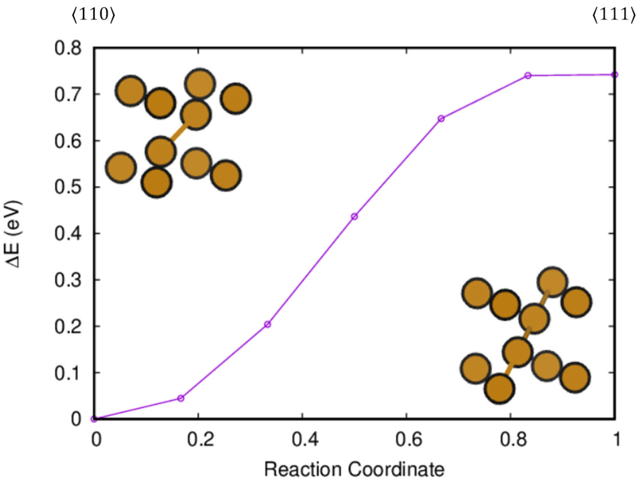

Figure 5. Nudged elastic band calculation between the ⟨110⟩ and ⟨111⟩ dumbbell self-interstitial atom configurations in FM BCC iron.

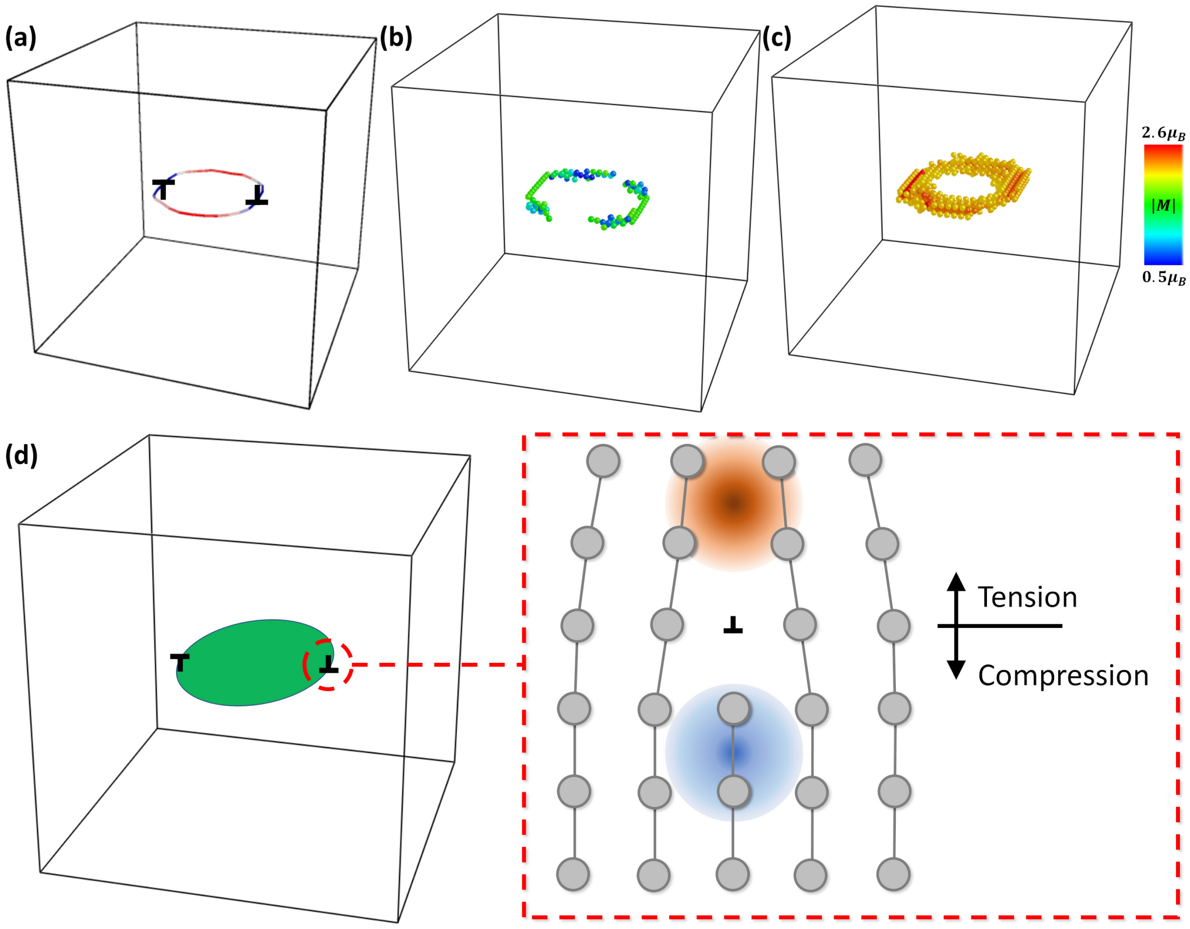

Figure 6. $\langle 100 \rangle$ mixed character dislocation loop in a $40 \times 40 \times 40$ (128,000 atom) simulation cell. Spin-lattice dynamics simulations were performed using SPILADY with the MSLP and relaxed by annealing. (a) Dislocation loop detected using the dislocation extraction algorithm (DXA) via OVITO showing the mixed screw (red) and edge (blue) character. Magnetic moments (b) smaller and (c) larger than bulk ($M_{\rm bulk} \approx 2.0\mu_B$). Only atoms whose moments satisfy $M \leq M_{\rm bulk} - 0.2\mu_B$ and $M \geq M_{\rm bulk} + 0.2\mu_B$, respectively, are shown. (d) Schematic of the dislocation loop and the atomic configuration of an edge dislocation indicating the compressed and tension regions.

The dislocation loop relaxed to be circular with mixed character as detected via the DXA routine in OVITO[33] and shown in Figure 6a. Atoms on the side of the edge type dislocation consisting of the extra half plane are under relative compression and are found to have their magnetic moments suppressed with magnitudes as small as half that of bulk (Figure 6b). On the other side which does not contain the extra half plane, the atoms are under tensile stress and are observed to have increased magnetic moments (Figure 6c). A simple schematic of the mixed character loop and the atomic distortions are shown in Figure 6d highlighting the regions under tension and compression relative to the orientation of the dislocation. The relationship between the local stress and magnitude of the magnetic moment is consistent with trends noted for strained bulk samples[29], as well as point defects and prismatic loops as shown in the main manuscript.

4 Code availability

The spin-lattice dynamics SPILADY code is available under the Apache Licence version 2.0. One can download it from https://ccfe.ukaea.uk/resources/spilady/. Version 1.0.1 is suitable for single element simulation of molecular dynamics, spin dynamics, spin-lattice dynamics and spin-lattice-electron dynamics[34]. It is capable of considering the longitudinal fluctuations of magnetic moments. The modified SPILADY code developed for this work, which includes the MSLP potential, is available upon request from the corresponding author and will be included in the next version release.

The interatomic potential fitting IPFIT code was developed in-house for this work. It is available upon request from the corresponding author subjecting to mutual agreements. Fitted parameters for our MSLP is provided as an additional file labelled *mslp.txt*. In Table 2, the keywords are identified in relation to the Hamiltonian of MSLP. We provide the equation number (in main manuscript) which defines the specific parameter.

It is to note SPILADY receives a more general form of the embedding function:

$$F = -\phi_0\sqrt{\rho} + \phi_1\rho^2 + \phi_2\rho^4. \tag{21}$$

This is equivalent to the embedding function defined in the Hamiltonian (Eq. 6 in main manuscript) where $\phi_0 = 1$, $\phi_1 = \phi$ and $\phi_2 = 0$.

References

1. Sonoda, S. & Murata, N. Neural network with unbounded activation functions is universal approximator. *Appl. Comput. Harmon. Analysis* **43**, 233 (2017).
2. Haley, P. & Soloway, D. Extrapolation limitations of multilayer feedforward neural networks. In *[Proceedings 1992] IJCNN International Joint Conference on Neural Networks*, vol. 4, 25–30 vol.4, 10.1109/IJCNN.1992.227294 (1992).
3. Miksch, A. M., Morawietz, J., T. Kästner, Urban, A. & Artrith, N. Strategies for the construction of machine-learning potentials for accurate and efficient atomic-scale simulations. *Mach. Learn. Sci. Technol.* **2**, 031001 (2021).
4. Smith, J. S., Nebgen, B., Lubbers, N., Isayev, O. & Roitberg, A. E. Less is more: Sampling chemical space with active learning. *J. Chem. Phys.* **148**, 241733 (2018).
5. Loeffler, T. D., Patra, T. K., Chan, H., Cherukara, M. & Sankaranarayana, S. K. R. S. Active learning the potential energy landscape for water clusters from sparse training data. *J. Phys. Chem. C* **124**, 4907 (2020).
6. Ma, P.-W. & Dudarev, S. L. Constrained density functional for noncollinear magnetism. *Phys. Rev. B* **91**, 054420 (2015).
7. Kresse, G. & Hafner, J. Ab initio molecular dynamics for liquid metals. *Phys. Rev. B* **47**, 558(R) (1993).
8. Kresse, G. & Hafner, J. Ab initio molecular-dynamics simulation of the liquid-metal–amorphous-semiconductor transition in germanium. *Phys. Rev. B* **49**, 14251 (1994).
9. Kresse, G. & Furthmüller, J. Efficient iterative schemes for ab initio total-energy calculations using a plane-wave basis set. *Phys. Rev. B* **54**, 11169–11186, 10.1103/PhysRevB.54.11169 (1996).
10. Kresse, G. & Furthmüller, J. Efficiency of ab initio total energy calculations for metals and semiconductors using a plane-wave basis set. *Comput. Mater. Sci.* **6**, 15 – 50 (1996).
11. Bain, E. C. & Dunkirk, N. Y. The nature of martensite. *Transactions Am. Inst. Min. Metall. Eng.* **70**, 25 (1924).
12. Derlet, P. M., Nguyen-Manh, D. & Dudarev, S. L. Multiscale modelling of crowdion and vacancy defects in body-centred-cubic transition metals. *Phys. Rev. B* **76**, 054107 (2007).
13. Ma, P.-W. & Dudarev, S. Langevin spin dynamics. *Phys. Rev. B* **83**, 134418 (2011).
14. Ma, P.-W., Woo, C. H. & Dudarev, S. L. Large-scale simulation of the spin-lattice dynamics in ferromagnetic iron. *Phys. Rev. B* **78**, 024434 (2008).

Table 2. Parameters in file *mslp.txt* for our iron MSLP

SPILADY Key	Parameter Symbol	Parameter details	Equation
V0	V_0	Neural network potential coefficient	16
a0	a_0	Landau A constant	14
a1	a_1	Landau A ρ coefficient	14
a2	a_2	Landau A ρ^2 coefficient	14
b0	b_0	Landau B constant	15
b1	b_1	Landau B ρ coefficient	15
b2	b_2	Landau B ρ^2 coefficient	15
c0	c	Landau C constant	13
phi0	ϕ_0	Embedding function $-\sqrt{\rho}$ coefficient	
phi1	ϕ_1	Embedding function ρ^2 coefficient (ϕ in Eqn 6)	6
phi2	ϕ_2	Embedding function ρ^4 coefficient	
J0	J	Pairwise Heisenberg exchange parameter	12
r1	r_{cut}	Cutoff length of Heisenberg exchange parameter	12
NV	N_V	Number of knots for the pair potential cubic spline potential	9
x1	r_1	Upper bound of ZBL potential. Start of interpolation region	9
x2	r_2	Lower bound of cubic spline potential. End of interpolation region	9
V0	V_0	Pair potential cubic spline knot parameter	9
V1	V_1	Pair potential cubic spline knot parameter	9
V2	V_2	Pair potential cubic spline knot parameter	9
V3	V_3	Pair potential cubic spline knot parameter	9
$\vdots$			
V(NV-1)	V_{NV-1}	Pair potential cubic spline knot parameter	9
rV0	r_0^V	Knot position for parameter V_0	9
rV1	r_1^V	Knot position for parameter V_1	9
rV2	r_2^V	Knot position for parameter V_2	9
rV3	r_3^V	Knot position for parameter V_3	9
$\vdots$			
r(NV-1)	r_{NV-1}^V	Knot position for parameter V_{NV-1}	9
Nt	N_t	Number of knots for the hopping integral cubic spline	8
t0	t_0	Hopping integral cubic spline parameter	8
t1	t_1	Hopping integral cubic spline parameter	8
t2	t_2	Hopping integral cubic spline parameter	8
$\vdots$			
t(Nt-1)	t_{Nt-1}		
rt0	r_0^t	Knot position for parameter t_0	8
rt1	r_1^t	Knot position for parameter t_1	8
rt2	r_2^t	Knot position for parameter t_2	8
$\vdots$			
rt(Nt-1)	r_{Nt-1}^t	Knot position for parameter t_{Nt-1}	8

SPILADY Key	Parameter Symbol	Parameter details	Equation
Nlayers	n	Number of layers of neural network (including input and output)	16-19
width0	$k=0$	Width of layer 0 (input layer) / Number of descriptors	16-19
width1	$k=1$	Width of hidden layer 1	16-19
$\vdots$			
widthK	$k=K=n-1$	Width of hidden layer K (K=n-1=output layer)	16-19
W1_0_0	$W^1_{0,0}$	Matrix element of weight between node 0 in layer 1 and node 0 in layer 0	16-19
W1_0_1	$W^1_{0,1}$	Matrix element of weight between node 0 in layer 1 and node 1 in layer 0	16-19
$\vdots$			
W1_1_0	$W^1_{1,0}$	Matrix element of weight between node 1 in layer 1 and node 0 in layer 0	16-19
$\vdots$			
Wk_i_j	$W^k_{i,j}$	Matrix element of weight between node i in layer k and node j in layer $k-1$	16-19
B1_0	b^1_0	Bias term for node 0 in layer 1	
B1_1	b^1_1	Bias term for node 1 in layer 1	
$\vdots$			
Bk_i	b^k_i	Bias term for node i in layer k	
r0	$R_s(x=0)$	Hyperparameter for 0th G2 descriptor	23
r1	$R_s(x=1)$	Hyperparameter for 1st G2 descriptor	23
$\vdots$			
rn		Hyperparameter for n^{th} G2 descriptor	23

15. Hohenberg, P. & Kohn, W. Inhomogeneous electron gas. *Phys. Rev. B* **136**, 864 (1964).

16. Kohn, W. & Sham, L. Self-consistent equations including exchange and correlation effects. *Phys. Rev. A* **140**, 1133 (1965).

17. von Barth, U. & Hedin, L. A local exchange-correlation potential for the spin polarized case. i. *J. Phys. C: Solid State Phys.* **5**, 1629 (1972).

18. Bl ochl, P. E. Projector augmented-wave method. *Phys. Rev. B* **50**, 1994 (1994).

19. Kresse, G. & Joubert, D. From ultrasoft pseudopotentials to the projector augmented-wave method. *Phys. Rev. B* **59**, 1758 (1999).

20. Hobbs, D., Kresse, G. & Hafner, J. Fully unconstrained noncollinear magnetism within the projector augmented-wave method. *Phys. Rev. B* **62**, 11556 (2000).

21. Perdew, J. P., Burke, K. & Ernzerhof, M. Generalized gradient approximation made simple. *Phys. Rev. Lett.* **77**, 3865 (1996).

22. Perdew, J. P., Burke, K. & Ernzerhof, M. Erratum: Generalized gradient approximation made simple. *Phys. Rev. Lett.* **78**, 1396 (1997).

23. Ma, P.-W. & Dudarev, S. L. Longitudinal magnetic fluctuations in langevin spin dynamics. *Phys. Rev. B* **86**, 054416 (2012).

24. Ma, P.-W. & Dudarev, S. L. *Atomistic Spin-Lattice Dynamics*, 1017–1035 (Springer International Publishing, Cham, 2020).

25. Ma, P.-W., Dudarev, S. L. & Wrobel, J. S. Dynamic simulation of structural phase transitions in magnetic iron. *Phys. Rev. B* **96**, 094418 (2017).

26. Chapman, J. B. J., Ma, P.-W. & Dudarev, S. L. Dynamics of magnetism in FeCr alloys with Cr clustering. *Phys. Rev. B* **99**, 184413 (2019).

27. Goryaeva, A. M., Maillet, J.-B. & Marinica, M.-C. Towards better efficiency of interatomic linear machine learning potentials. *Comput. Mater. Sci.* **166**, 200 (2019).

28. Cooper, A. M., Kästner, J., Urban, A. & Artrith, N. Efficient training of ann potentials by including atomic forces via taylor expansion and application to water and a transition-metal oxide. *npj Comput. Mater.* **6**, 54 (2020).

29. Chapman, J. B. J., Ma, P. W. & Dudarev, S. L. Effect of non-heisenberg magnetic interactions on defects in ferromagnetic iron. *Phys. Rev. B* **102**, 224106 (2020).

30. Olsson, P., Domain, C. & Wallenius, J. Ab initio study of Cr interactions with point defects in bcc Fe. *Phys. Rev. B* **75**, 014110 (2007).

31. Fu, C. C., Willaime, F. & Ordejón, P. Stability and mobility of mono- and di-interstitials in α-Fe. *Phys. Rev. Lett.* **92**, 175503 (2004).

32. Hirel, P. Atomsk: A tool for manipulating and converting atomic data files. *Comput. Phys. Commun.* **197**, 212 (2015).

33. Stukowski, A. Visualization and analysis of atomistic simulation data with ovito – the open visualization tool. *Model. Simul. Mater. Sci. Eng.* **18**, 015012 (2020).

34. Ma, P.-W., Dudarev, S. L. & Woo, C. H. Spilady: A parallel cpu and gpu code for spinlattice magnetic molecular dynamics simulations. *Comput. Phys. Commun.* **207**, 350 (2016).
